# Household crowding and mortality before and during the COVID-19 pandemic among adults: Findings from longitudinal population surveillance data in rural and peri-urban settings in Limpopo, South Africa

**DOI:** 10.1186/s12963-025-00391-z

**Published:** 2025-10-30

**Authors:** Kagiso Peace Seakamela, Jean Juste Harrisson Bashingwa, Joseph Tlouyamma, Cairo Bruce Ntimana, Modupi Peter Mphekgwana, Reneilwe Given Mashaba, Katlego Mothapo, Chodziwadziwa Whiteson Kabudula, Eric Maimela

**Affiliations:** 1 DIMAMO Population Health Research Centre, University of Limpopo, Sovenga 0727, Polokwane, 0700 South Africa; 2 MRC/Wits Rural Public Health and Health Transitions Research Unit (Agincourt), Faculty of Health Sciences, School of Public Health, University of the Witwatersrand, Johannesburg, South Africa; 3 Department of Computer Science, University of Limpopo, Sovenga 0727, Polokwane, 0700 South Africa; 4 Research Administration and Development, University of Limpopo, Sovenga 0727, Polokwane, 0700 South Africa; 5 Department of Public Health, University of Limpopo, Sovenga 0727, Polokwane, 0700 South Africa

**Keywords:** Household crowding, Age, Gender, Mortality rates, COVID-19, Africa

## Abstract

**Background:**

Household overcrowding is a public health concern linked to increased morbidity and mortality. There is limited data available on the effects of COVID-19 on age-specific mortality in the context of household crowding in rural and peri-urban settings in Africa. Here we assess age-specific excess mortality in densely inhabited households before and during COVID-19.

**Methods:**

We used data collected three times annually between 2019 and 2021 in the health and demographic surveillance project in DIMAMO, South Africa. Data inaccuracies or inconsistencies were identified and corrected using data validation rules or algorithms implemented at both application and database levels. The number of persons-per-room was used to determine the degree of crowding or household crowding index (HCI). HCI tertiles were categorized as low, medium, and high density.

**Results:**

Throughout the study, people aged 70 years and above had the highest mortality rates compared to other age groups (40–54 and 55–69), with the highest mortality rates observed in overcrowded households (highest crowding index). MGH was observed as a risk factor for mortality during COVID-19. Individuals aged 70 years and older had the highest hazard ratios before and during COVID-19, where the risk increased during COVID-19 for densely populated households.

**Conclusion:**

Overcrowding at the household level was associated with increased mortality during COVID-19 for individuals aged 70 years and older. Public health interventions in the case of future pandemics should consider how to address this risk factor.

**Supplementary Information:**

The online version contains supplementary material available at 10.1186/s12963-025-00391-z.

## Introduction

The COVID-19 pandemic caused by the SARS-CoV-2 (severe acute respiratory syndrome coronavirus) virus outbreak has been one of the public health concerns impacting approximately 5,4 million people worldwide by 2021 [1–3]. Overcrowding was an important risk factor during the COVID-19 pandemic (4). Health institutions of most nations adopted lockdowns to reduce movement and overcrowding in areas that could be classified as a hotspot [4–9].

Household overcrowding is defined as more than two people per room (excluding the bathroom but including the kitchen and the living room [10]. Household overcrowding is a public health concern both locally and internationally [7, 11, 12]. Household overcrowding is associated with COVID-related all-cause mortality. Literature reported that densely populated households have had more deaths during COVID-19 [13, 14].

The overcrowded households develop interior moisture and dampness which has been reported to create a conducive environment for pests, mold, and viruses to grow and spread [15]. Household overcrowding is also a concern in South Africa. Nkosi and colleagues reported a prevalence of overcrowding to be 57.6% in the two suburban areas of Johannesburg, wherein they found an association between household overcrowding with acute respiratory, and gastrointestinal symptoms and fever/chill [16]. Households that younger individuals mostly occupy might not experience mortality due to overcrowding compared to those occupied by elderly people. Hence, some studies have reported no relationship between household size and all-cause mortality during the pandemic [17].

Households in rural areas are mostly occupied by youth and elderly people/pensioners. The elderly people are mostly taking care of their grandchildren while their parents migrate to cities for better opportunities (employment and education) [18, 19]. The existence of more than one generation in a household called Multigenerational households, brings about a different risk of mortality due to COVID-19 [20, 21]. The younger populations which are mobile could increase the risk of elderly people contracting COVID-19. Elderly people were more at risk of suffering from adverse complications of COVID-19 which included hospitalization and death due to morbidities, ageing and socioeconomic status [22].

Studies that have assessed COVID-19 vulnerability reported a higher risk in individuals who are single, older and those with low socioeconomic status [23–25]. The household overcrowding index which only accounts for the number of people in a household and the number of rooms, does not account for the age groups that dwell in a household [26, 27]. Using only the household overcrowding index to estimate the impact of COVID-19 on all-cause mortality during the pandemic might not reflect the true reality. In South Africa, the study by Nkosi et al. [16] investigated the effect of household size on mortality during COVID-19. The study by Nkosi et al. [16] was conducted in an urban setting, using a cross-sectional design, without vising the household.

Literature suggests that densely populated households were at a higher risk of contracting COVID-19 and high all-cause mortality [9]. However, little was done to investigate the age-specific mortality within densely populated households during COVID-19. Determining if density has a substantial role in explaining the death rates during COVID-19 has significant ramifications for socioeconomic development and public policy [28]. This study investigates and evaluates the effect of densely inhabited households on age-specific death rates during the COVID-19 period. The current study also investigates the effect of multigenerational households on the likelihood of mortality during COVID-19 in overcrowded households.

## Methodology

### Background

This study was based on data obtained from Dikgale, Mamabolo, and Mothiba tribal, which collectively make up the DIMAMO Health Demographic Surveillance Systems (HDSS). The HDSS was established in 1995 and is situated in the Capricorn District of Limpopo Province, South Africa. The surveillance area consists of 59 villages, which are classified into rural and semi-rural areas. These areas are inhabited by people of low socioeconomic status with high levels of unemployment, poor educational backgrounds, and a high prevalence of hypertension and other chronic illnesses. Since its establishment, the DIMAMO surveillance area has undergone two major expansions, which have tremendously increased the population under surveillance. The expansions were in 2010 and 2018, with populations of approximately 34,000 and 100,000, respectively. The current population is over 100,000 from approximately 21,000 households.

### Study design

Leveraging the HDSS platform, this study used data collected through a longitudinal survey approach to investigate the phenomenon of interest. The longitudinal nature of the data makes it possible to examine patterns and track changes over an extended period. The HDSS location was meticulously chosen to guarantee its variety and representation of the target population.

### Data collection

We used demographic and socioeconomic status data prospectively collected from 2015 to 2021 within the DIMAMO Health and Demographic Surveillance Site. The HDSS collects its data primarily through household surveys. Household surveys collect data on demographic factors, health status, healthcare usage, and other variables of interest. Data was collected by trained field workers. Data collection is done in three levels viz. dwelling, household, and individual. Each household and individual are allocated a unique identifier to enable linkage across survey cycles. The sample size is routinely revised to account for population changes, and attempts are taken to reduce non-contact rates to maintain the integrity of the dataset.

The triannual surveillance approach was used to ensure complete data coverage and quarterly updates of recent events. The approach enabled three data collection points in one census round viz. one physical visit to the households and two points of telephonic data collection. In physical data collection, fieldworkers directly visit households to update or register households. There is also telephonic data collection conducted by call centre agents to update household data. This approach became advantageous during the COVID-19 era with government restrictions in place. The data was collected without physically visiting the households, which allowed the continued update of data. However, households without contact numbers could not have their data updated, especially during COVID-19. Post COVID-19 pandemic the centre focused on updating household contact numbers to ensure continued data collection in case of future pandemics.

### Data management

Data management is an essential component of HDSS research to maintain the quality and integrity of data. The survey data was collected electronically using the Survey Solutions data collection platform and permanently stored in the MS SQL server database. Data inaccuracies or inconsistencies were identified and corrected using data validation rules or algorithms implemented at both application and database levels. The algorithms ensured data consistency, completeness, validity, and quality during the collection. The data quality was improved by using stored procedures in the database to check the data before saving it to the MS SQL server. The data was pre-processed to the required format using the Pentaho data integration tool and R studio for the analysis [29, 30].

### Data preparation

We assessed how the number of rooms in each household changed over the years. The rate of change in the number of rooms per household and year was low (< 3%), which allowed us to impute data where necessary using the most recent information available in household data backwards. The household crowding index (HCI) was used to determine the degree of crowding in a household. The HCI was calculated by dividing the number of people residing in the household (per year) by the number of rooms (per year) and then categorized into tertiles namely T1, T2 and T3 which represent low HCI, Medium HCI and higher HCI respectively.

Additionally, a new variable called “Multigenerational households” (MGH) was created to detect the co-existence of elderly people (> 39 years) and younger persons (5–39 years) in the same household at a given time. Two variables were created: MGH with 5–24 (elder people residing with younger aged 5–24 years) and MGH with 25–39 (elderly people residing with younger aged 25–39 years). Finally, we restricted the data to people aged 40 years and above at any time for this study because the effect of COVID-19 was most likely to be seen in elderly populations.

### Statistical analyses

We generated frequencies (person-years and the number of deaths) for age group, sex, crowding index, and year. Survival was calculated using the Kaplan–Meier estimator. Patients who remained without the event by the end of the period 2021 were censored. The Cox Proportional hazard model was used to estimate the Hazard ratio for death with a corresponding 95% confidence interval for each socioeconomic and demographic factor. We assessed a two-way interaction between each predictor and crowding index variable, and a three-way interaction between age group, crowding index, and period, i.e., the period before COVID-19 (2015–2019) and during COVID-19 (2020–2021). Univariate and multivariate Cox models were tested.

## Results

Table 1 between 2015 and 2018, the crude mortality rate among the DIMAMO HDSS declined. However, from 2019 through 2021, we saw an increase in the crude mortality rate with males having the highest crude mortality rates. Throughout the study period, people aged 70 and older had the highest mortality rate than any other age group in the HDSS. The mortality rate among people aged 70 years and older increased by 46.8 (per 1000 person-years) from 2015 (52.3) to 2021 (99.10). The mortality rate among households with low and high crowding index increased from 15.7 to 40.4 and 25.3 to 32.87 respectively, between 2015 and 2021. Mortality rates increased for elderly people living with younger persons during the COVID-19 period (2019–2021) with MGH of elderly and individuals aged 25–39 recording the highest mortality rates.

**Table 1:** Crude mortality (per 1000 people) from 2015 to 2021 among DIMAMO HDSS by social demographic characteristics

	2015	2016	2017	2018	2019	2020	2021
CMR	22.54	21.93	21.39	17.18	20.92	24.20	35.97
Gender							
Female	20.64	18.59	20.65	15.91	18.32	21.77	35.48
Male	25.28	26.67	22.43	18.98	24.68	27.73	36.71
Age Category							
40–54	10.46	11.99	9.62	7.18	9.24	9.85	11.33
55–69	24.07	18.18	22.38	15.52	20.89	20.23	34.17
70 +	52.26	55.10	50.96	45.93	52.53	68.58	99.10
Household Crowding							
Low	15.68	20.95	21.30	18.87	21.39	29.92	40.36
Medium	23.83	24.14	21.39	18.19	21.27	22.53	35.47
High	25.33	20.59	21.44	15.12	20.29	21.87	32.84
Multigenerational households5-24							
No	22.08	26.30	25.53	21.70	26.29	32.39	42.85
Yes	22.64	20.94	20.38	15.94	19.42	21.82	33.73
Multigenerational households25-39							
No	18.15	20.07	17.57	18.44	20.61	26.58	33.98
Yes	24.17	22.66	22.88	16.65	21.05	23.18	37.01

Table 2 crude mortality rates amongst the gender category overall were highest in the lower crowding index, with more males dying across all the household indexes than females. Throughout the study, people aged 70 years and older had the highest mortality rates compared to other age groups (40–54 and 55–69), with the highest mortality rates observed in overcrowded households (highest crowding index).

**Table 2:** Crude Mortality rate (per 1000 people) and household crowding index by sociodemographic characteristics

	Low HCI		Medium HCI		High HCI	
	CMR	Person-years	CMR	Person-years	CMR	Person-years
Gender						
Female	27.5	15,912	22.34	23,858	20.55	27,012
Male	26.46	13,756	27.97	15,661	27.21	16,686
Age category						
40–54	8.44	11,736	10.03	20,131	10.62	23,349
55–69	22.98	10,095	23.82	12,302	22.85	14,089
70+	60.10	7837	67.17	7086	70.13	6260
MGH (5–24)						
Yes	22.01	10,951	23.06	34,469	23.07	42,916
No	29.97	18,717	34.86	5049	24.27	783
MGH (25–39)						
Yes	26.45	11,117	24.98	29,343	24.08	38,284
No	27.38	18,551	23.39	10,175	16.07	5415

**Fig. 1:**
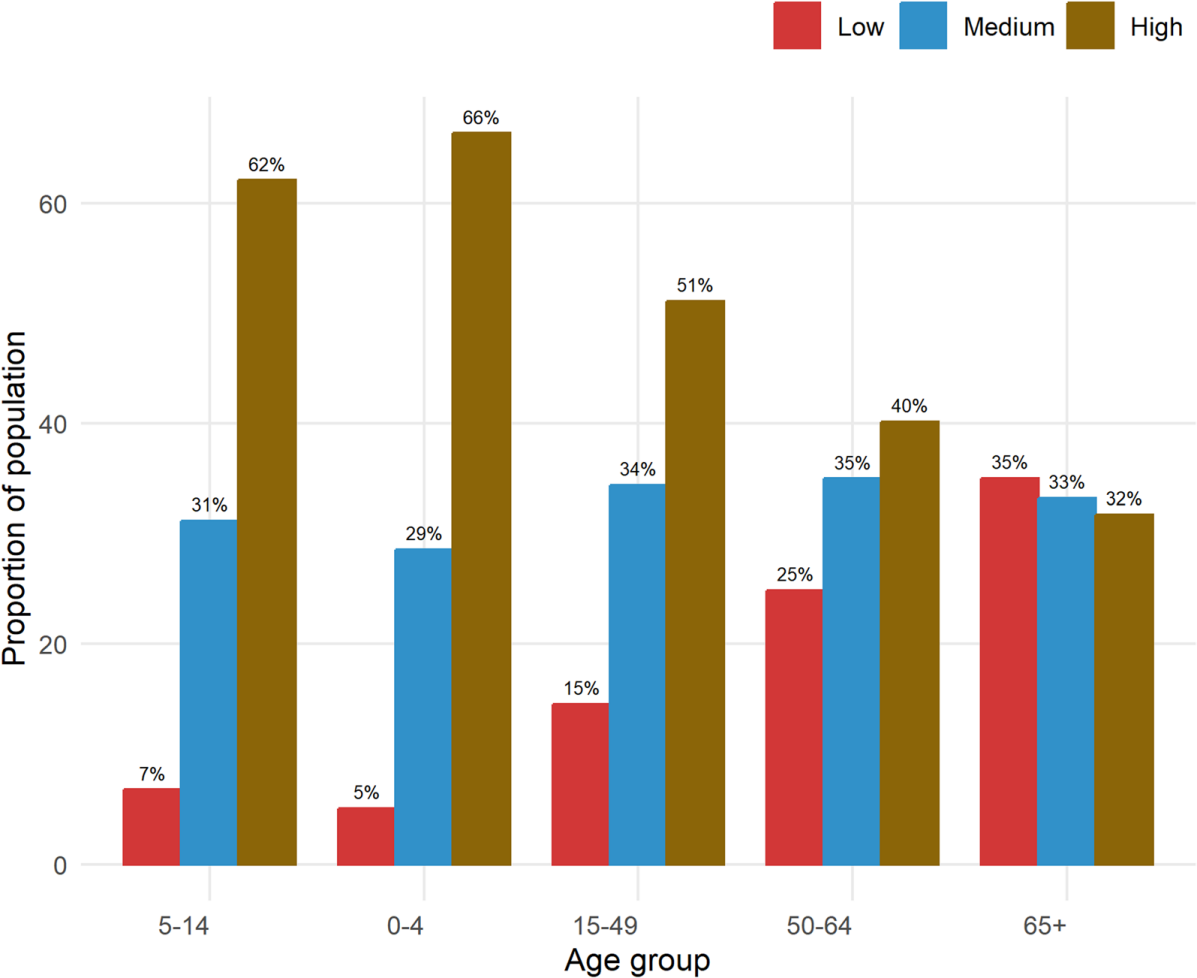
The proportion of people per age group and household crowding index

**Fig. 2:**
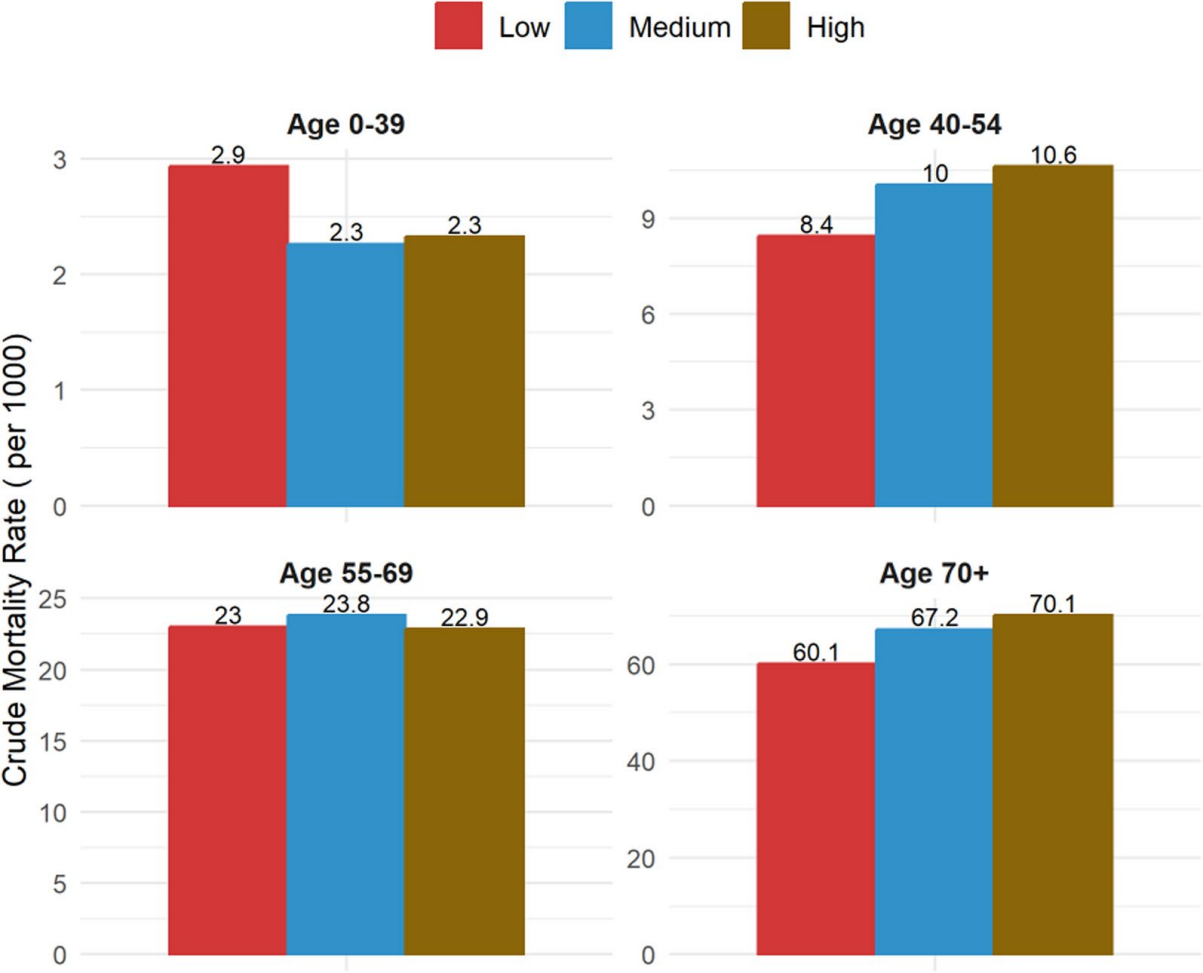
Crude mortality rates by age groups and household crowding index

Figure 1 provides a snapshot of the distribution of various sex-specific age groups in categories that define household crowding. It is evident from the figure that as age increases, the proportion of the population also increases in households with a low crowding index, meaning the higher proportions of elderly people (65 and above) are associated with a low household crowding index. Furthermore, as shown in Fig. 2, crude mortality rates are high for elderly populations aged 40–54 and 70 + , with the highest crude mortality rate observed in the 70 + age group.

All the variables except for MGH (25–39 years) were found to be significantly associated with the risk of mortality in the DIMAMO HDSS, as shown in Table 3. Males had a higher risk of mortality as compared to females (1,43; 1,54–1,32CI). The age group 70 years and older was 7 times more likelihood of mortality as compared to younger age groups (40–54 years). People staying in medium and highly crowded households were 20% more likely to die as compared to low-crowded households (1.20; 1.35–1.06CI vs 1.24; 1.41–1.09CI respectively). In terms of younger and older co-residents, it has been found that MGH with the younger age group between 5 and 24 years decreased the chance of dying among elderly persons aged 40 and above.

**Table 3:** The association between risk of mortality, household crowding index and sociodemographic factors

	HR	LowerCI	UpperCI	*p*.value
Period				
During COVID-19	1.43	1.32	1.54	< 0.001
Gender				
Male	1.49	1.38	1.60	< 0.001
Age category				
55–69	2.37	2.13	2.64	< 0.001
70 +	7.09	6.41	7.84	< 0.001
Household crowding				
Medium	1.20	1.06	1.35	< 0.001
High	1.24	1.09	1.41	< 0.001
MGH				
MGH (5–24 years)	0.87	0.78	0.97	0.010
MGH (25–39 years)	1.05	0.95	1.15	0.360

### Significant p-value ≤ 0.05, non-significant p-value ≥ 0.05, HR = Hazard ratio

**Fig. 3:**
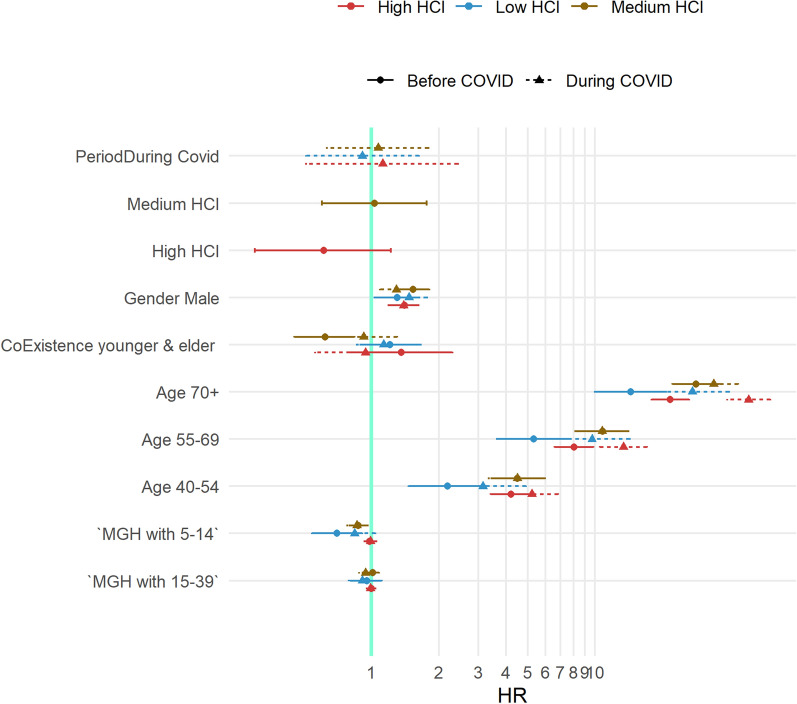
Cox regression illustrates fluctuations in hazard ratios, before COVID-19 and during COVID-19 in terms of gender, household crowed index, MGH, and age categories

Figure 3 it was observed that MGH with people in the younger age group (between 5 and 24 years) increased the risk of dying among elderly persons aged 40 and above who are living in highly crowded households during COVID-19 (2, 1-4CI). Older individuals aged 70 and above had the highest risk of dying before and during COVID-19, differentials in estimated hazard ratios between the two periods were observed, with the highest risks of mortality in densely populated households during the pandemic period (7HR during COVID-19 vs 5HR before COVID-19).

## Discussion

This study investigated and evaluated the effect of densely inhabited and MGH on age-specific death rates during the COVID-19 period. Mortality rates varied over the years at the DIMAMO HDSS with an increase in trend from 2019 to 2021. Males had the highest mortality rates compared to females throughout the study. Individuals aged 70 and above residing in households with higher crowding index were 7 × more likely to experience mortality as compared to the younger elderly group (40–54 and 55–69).

In terms of gender, males had the highest mortality rates compared to females over the years (before and during COVID-19). Males were more at risk of mortality with the highest hazard ratios recorded in a household with the highest crowding index. The findings of the present study concur with findings from another study that reported high mortality rates in men as compared to women [31–33]. This may be because men are less health conscious and engage in lifestyle risk factors i.e. smoking and alcohol consumption compared to women as well as presenting late to the hospital when sick [31, 34, 35].

There was a general decrease in death rates across all variables from 2015 to 2018 and a sharp increase during COVID-19 in 2020–2021. As with our findings, other studies have indicated higher than usual mortality rates among individuals aged 65 and above during the COVID-19 pandemic [30, 32–34]. Factors associated with increased risk of mortality among older individuals during COVID-19 are age-related and overlapping non-communicable diseases such as hypertension, obesity, diabetes and age-related immune function loss [30, 35]. Older individuals living in households with a higher crowding index had the highest mortality rates during COVID-19 compared to less-crowded households, as reported in other studies that assessed the effect of the COVID-19 pandemic on all-cause mortality [7, 36].

Participants aged 70 and above who were living in a household with a high crowding index had a higher risk of death during COVID-19 compared to those in less-crowded households. In accordance with the current study, Hills and Eraso reported that participants aged 70 and up who lived in crowded households were more vulnerable to COVID-19 [37]. Multigenerational households were also a risk factor for mortality during COVID-19. Older individuals who were residing with younger individuals (5–24) in a household with a higher crowding index had higher hazard ratios of mortality during COVID-19 compared to before COVID-19. Similarly, Brandén and colleagues reported older individuals who are residing with individuals who are of working age increased the risk of mortality during COVID-19 [38]. The younger age groups that are more mobile could contract the SARS-CoV-2 coronavirus and expose the elderly at home when they return home hence increasing the risk of mortality for older individuals during COVID-19.

Also, the high household crowding index had higher hazard ratios compared to the lower household crowding index. Overcrowding is linked to an increased risk for several respiratory illnesses [16, 39, 40], and the proximity of individuals in overcrowded residences was reported to promote the transmission of COVID-19 [41–43]. Overcrowded households are linked to poverty and poor access to health services [44].

Because of the simultaneous risk of overcrowding on both underlying health conditions, poverty, poor health access and the spread of infectious diseases, communities with high rates of household crowding may also have higher rates of COVID-19 transmission and mortality [40].

## Conclusion

This is one of the first studies to assess the effect of household crowding on all-cause mortality during COVID-19 in sub-Saharan Africa. In the DIMAMO HDSS, household overcrowding was associated with increased all-cause mortality during the COVID-19 pandemic. Overlapping sociodemographic factors like being elderly (> 65), male, and in highly crowded housing increased the risk of all-cause mortality during the COVID-19 pandemic in the African population of Limpopo Province, South Africa. This study demonstrates that the DIMAMO HDSS data can be used to answer timely population and household-level questions, which can be used in resource distribution and rural development by identifying populations at risk in the case of future pandemics.

## Supplementary Information

Supplementary material 1

## Abbreviations

TREC: Turfloop research ethics committee
HDSS: Health demographic surveillance system
HCI: High crowding index
SARS-CoV2: Severe acute respiratory syndrome coronavirus 2
MGH: Multigenerational household
HR: Hazard ratios
SAPRIN: South Africa population research infrastructure network
